# Nomenclatural stability and the longevity of helminth species names

**DOI:** 10.1007/s11230-024-10161-4

**Published:** 2024-05-03

**Authors:** Robert Poulin, Bronwen Presswell

**Affiliations:** Department of Zoology, University of Otago, PO Box 56, Dunedin, New Zealand

## Abstract

Although most Latin binomial names of species are valid, many are eventually unaccepted when they are found to be synonyms of previously described species, or superseded by a new combination when the species they denote are moved to a different genus. What proportion of parasite species names become unaccepted over time, and how long does it take for incorrect names to become unaccepted? Here, we address these questions using a dataset comprising thousands of species names of parasitic helminths from four higher taxa (Acanthocephala, Nematoda, Cestoda, and Trematoda). Overall, among species names proposed in the past two-and-a-half centuries, nearly one-third have since been unaccepted, the most common reason being that they have been superseded by a new combination. A greater proportion of older names (proposed pre-1950) have since been unaccepted compared to names proposed more recently, however most taxonomic acts leading to species names being unaccepted (through either synonymy or reclassification) occurred in the past few decades. Overall, the average longevity of helminth species names that are currently unaccepted was 29 years; although many remained in use for over 100 years, about 50% of the total were invalidated within 20 years of first being proposed. The patterns observed were roughly the same for all four higher helminth taxa considered here. Our results provide a quantitative illustration of the self-correcting nature of parasite taxonomy, and can also help to calibrate future estimates of total parasite biodiversity.

**Supplementary Information:**

The online version contains supplementary material available at 10.1007/s11230-024-10161-4.

## Introduction

Many recent analyses and commentaries have focused on the Latin binomial names of species, specifically on their etymology and even the potential consequences of a chosen name for the future study of a species (e.g., Poulin et al. 2022; Mammola et al. 2023; Mlynarek et al. 2023; Heard and Mlynarek 2023). Beyond the inspiration for a species name, its long-term retention among accepted names within taxonomic and biodiversity databases also matters. In accordance with the rules of the International Code of Zoological Nomenclature, or ICZN (https://www.iczn.org), there are multiple reasons why a Latin binomial name might eventually be invalidated and no longer accepted. For example, a species name can be synonymised if the species it denotes is found to be equivalent to an earlier name. In other words, the two names refer to the same biological species, and only the earlier name is considered valid; the other name becomes unaccepted. The proportion of all species names within any given higher taxonomic group that are synonyms can be very high, exceeding 20% in some cases (Solow et al. 1995). Such high numbers of invalid Latin names that do not represent distinct species have important consequences: they complicate attempts to estimate biodiversity (Alroy 2002) as well as literature searches for information about particular species (Guala 2016). Another common reason why a Latin binomial name may no longer be accepted is when the species it denotes is moved to a different genus following a careful taxonomic re-assessment of its classification. In these cases, the new classification stands and the new binomial name supersedes the older one, which is no longer accepted.

Several dubious taxonomic practices have caused the proliferation of new species names that eventually become unaccepted. These include ‘taxonomic vandalism’, which consists of using trivial morphological variation as an unjustifiable basis to erect a new species (Wüster et al. 2021); ‘nomenclatural mihilism’, whereby authors seek to secure recognition and a place in posterity by naming new species with little or no biological justification (Dubois 2008; Evenhuis 2008); and ‘nomenclatural harvesting’, which consists of naming apparent taxonomic units identified from phylogenies published by other researchers, but without studying actual physical specimens (Denzer and Kaiser 2023). These practices unjustifiably inflate the number of species names, causing headaches for taxonomists who later have to sort out the mess. If Latin names proposed through these practices are not later synonymysed with existing species names or superseded by a different name following a taxonomic re-classification, they may persist, but with an uncertain taxonomic status casting doubt over their validity.

Most species names are valid, of course, and represent distinct species. However, in the case of species names that are synonyms of earlier ones or that require re-naming because the species they denote belongs to a different genus, how long does it take for them to become officially unaccepted? In other words, what is the longevity of invalid species names? Because assessing the validity of existing species names requires careful work and because newly-discovered species keep taxonomists occupied, it can take years following the publication of a Latin species name before, if deemed necessary, it is invalidated and unaccepted. We might therefore expect a higher proportion of unaccepted names among those erected many years ago than among those coined more recently; is this the case? Here, we address these questions for large subsets of parasite species from each of four higher helminth taxa (Acanthocephala, Nematoda, Cestoda, and Trematoda). We assess differences in nomenclatural stability both among these taxa and over time, and provide the first quantitative assessment of the frequency at which Latin binomial names are unaccepted as well as how long it takes for incorrect names to become unaccepted.

## Methods

The WoRMS database (World Register of Marine Species; https://www.marinespecies.org/) was used as a primary source of data. Although biased toward marine species, its content is controlled and checked by taxonomic experts, and unlike other databases it provides information on synonymy and historical changes in the validity of species names. We downloaded species data from WoRMS in mid-November 2023. The data needed to be manually curated prior to our analyses, with some species names requiring additional literature searches; due to these time-consuming factors, we did not include all existing species names but instead only a large representative subset. We first downloaded all species names of acanthocephalans, as this is the least speciose taxon of the four considered here. Since acanthocephalan species numbered a little over 1000 names, we adopted the following approach for the other three helminth taxa to obtain roughly comparable numbers. We ranked families of trematodes, cestodes and nematodes from most to least speciose, separately for each of the three taxa, based on entries in WoRMS. We then included families, starting with the most speciose, until our running total of species surpassed 1000, but with at least three families included per taxon (Table 1). Although far from covering all species in those three higher taxa, the large datasets generated by this method nevertheless allow meaningful estimates of nomenclatural stability.

**Table 1:** Numbers of species names of different taxonomic status for each of the four higher helminth taxa

Helminth taxon	Families included	Uncertain	Unaccepted (longevity unknown)	Unaccepted (longevity known)	Accepted	Total
Acanthocephala	All	15	28	391	801	1235
Nematoda	Cucullanidae, Raphidascarididae, Camallanidae, Anisakidae, Cysticolididae	119	58	370	791	1338
Cestoda	Onchobothriidae, Proteocephalidae, Hymenolepididae	49	25	284	771	1129
Trematoda	Opecoelidae*, Microphallidae, Gorgoderidae	43	52	526	1059	1680

*Only genera with names beginning with A to P

We pruned the lists of species names by deleting names considered invalid simply because of a spelling error or an incorrect Latin suffix (denoted as ‘lapsus’ or ‘malformed suffix’ in WoRMS). Correction of a name that was originally misspelled is not a taxonomic act, and does not invalidate the taxonomic intent of its authors. In such cases, we retained only the correct version as this was always included in WoRMS as a separate entry. This left only species names with taxonomic status classified as accepted, unaccepted, or uncertain (the latter including ‘nomen dubium’, ‘nomen nudum’, or ‘taxon inquirendum’). Unaccepted Latin binomial names fell into three categories: (i) superseded combination, when a species was moved to a different genus in the years following its original description and naming; (ii) synonym, when a species has been synonymised with (or reduced to a junior homonym or subspecies of) a previously described species whose earlier name takes precedence; and less frequently (iii) pre-occupied, when a species name was later found to have previously been given to a different species, requiring re-naming of the more recently discovered species. The year in which a species name was invalidated (i.e., superseded, synonymised, or found to be pre-occupied) was usually obtainable from WoRMS. However, in many cases additional searches of individual species names in the scientific literature were required to obtain the year when species names were invalidated. Because searches often proved fruitless after several minutes, and because of the large number of unaccepted names for which a literature search was necessary, we could not obtain the year in which a species name was invalidated in all cases. The entire final dataset is available as Supplementary Material to this article.

Our analyses are mostly exploratory in nature, not hypothesis-driven. Therefore, we use qualitative and visual overviews rather than outputs of statistical tests; our dataset is freely available to anyone wishing to explore it in greater detail. First, we compared the most frequent reasons why certain species names are no longer accepted, among the four helminth taxa. For this, we grouped unaccepted names, separately for each helminth taxon, into the three categories based on the reason they are no longer accepted, i.e., superseded, synonymised, or found to be pre-occupied. We then contrasted the relative frequencies of these reasons for invalidating a species name among the four helminth taxa.

Second, we compared the proportion of species names that are no longer accepted among the four helminth taxa and also among the different time periods in which the names were originally proposed. For this, in order to have sufficient numbers of species per time period, we defined time periods as follows: pre-1900, 1901–1925, 1926–1950, 1951–1975, 1976–2000, and post-2000. The null expectation is that names proposed in earlier periods would include a higher proportion that have since become unaccepted, since there has been more time to reassess their validity, with no difference expected among higher helminth taxa.

Third, we compared the average longevity of species names that are no longer accepted among the four helminth taxa and also among the different time periods in which the names were originally proposed. For this, we used the same time periods as above. The longevity of a species name was calculated as the number of years between the year when it was originally proposed and the year when it became unaccepted. This resulted in some zero values, where a species name apparently became unaccepted in the same year that it was coined. It also produced a small number of negative values, even after double-checking the corresponding entries in our dataset; because negative values would indicate that a species name was invalidated before it was even first proposed, they are obviously errors whose source could not be identified, and they were excluded from our longevity estimates. For the longevity data, the null expectation is that names proposed in earlier times that are no longer accepted should have had a greater longevity than those proposed more recently, with no difference expected among higher helminth taxa.

## Results

Overall, our study included 5382 Latin binomial names of helminth species. The numbers of uncertain, unaccepted and accepted species names for each of the four helminth taxa are given in Table 1. The oldest species name included in our study was coined in 1767, while the number of species names originating in each time period was generally highest in either the middle or the most recent time periods.

**Fig. 1:**
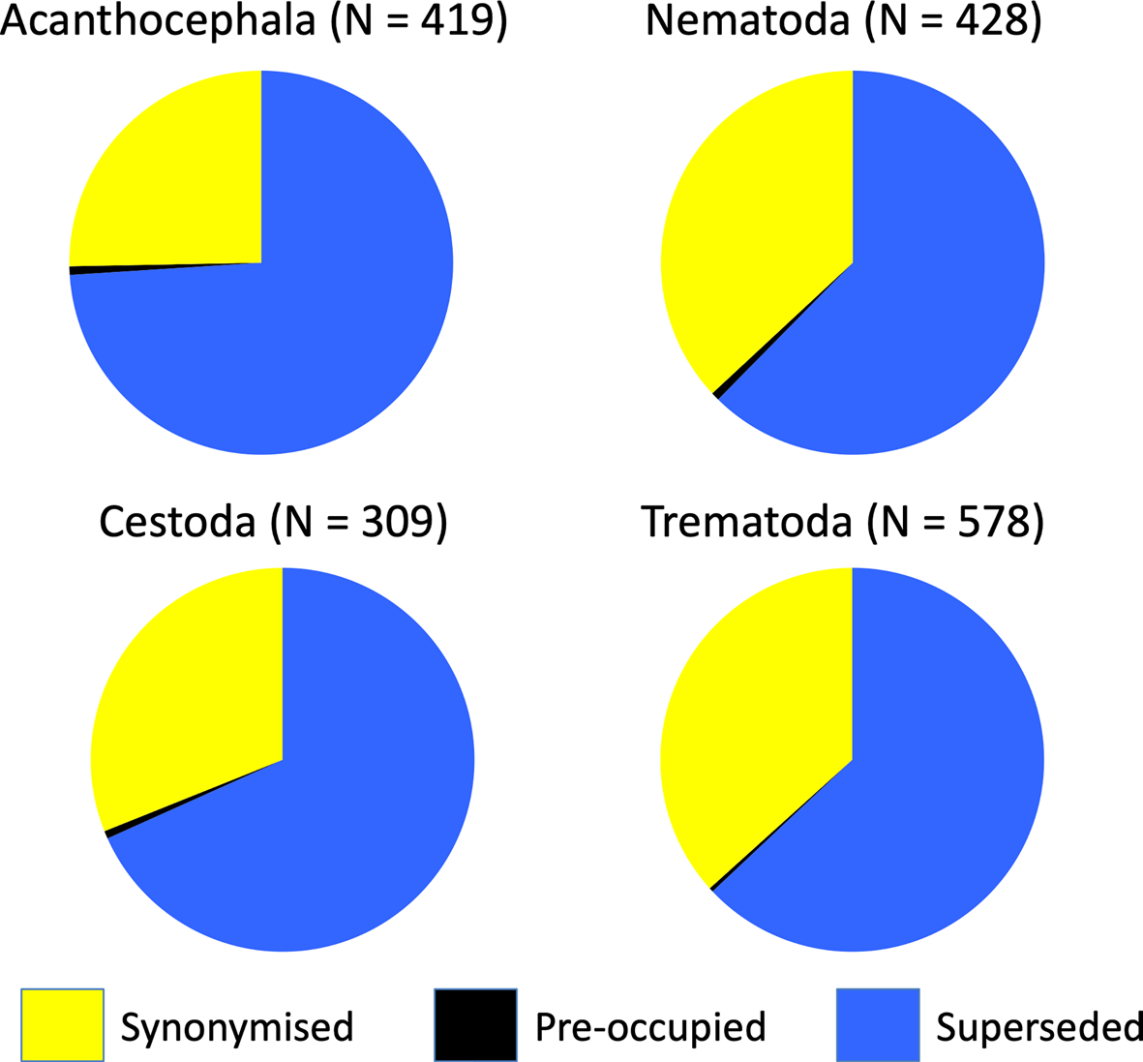
Relative frequencies of the reasons given for Latin binomial names of species to become unaccepted: the names are either synonymised with an earlier name, pre-occupied by having been given earlier to another species, or superseded by a new binomial combination when the species is subsequently transferred to a different genus. Data are shown separately for each of the four higher taxa of helminth parasites. Sample sizes shown include only unaccepted names

The most common reason why Latin species names became unaccepted was because they were superseded by a different binomial combination, followed by synonymisation with another species name; very few names were unaccepted because they were found to be pre-occupied (Figure 1). The patterns were very similar among the four helminth taxa, with superseded combinations accounting for two-thirds to three-quarters of unaccepted names.

**Fig. 2:**
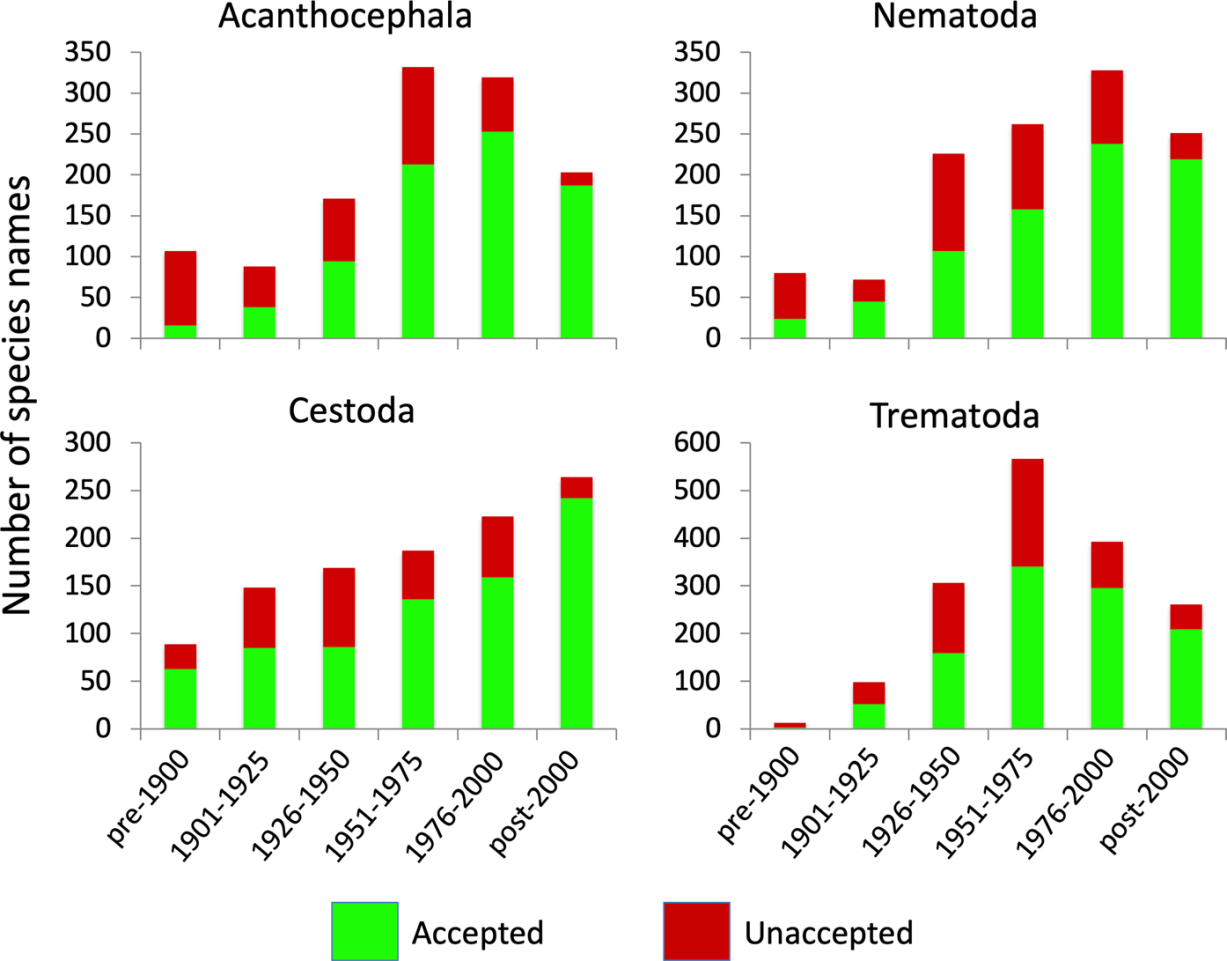
Relative frequencies of Latin binomial names of species described in each time period that are either still valid and accepted, or that later became unaccepted and are no longer valid. Data are shown separately for each of the four higher taxa of helminth parasites, and according to the time period in which species names were first coined

The proportion of all species names that subsequently became unaccepted was roughly similar among the four helminth taxa; overall, it was highest for trematodes (34.4% overall) and lowest for cestodes (27.4%). However, the proportion of species names that were eventually unaccepted varied among time periods (Figure 2). It was generally higher for older names, i.e. those proposed in the earlier time periods, although this pattern is not so pronounced for cestodes.

**Fig. 3:**
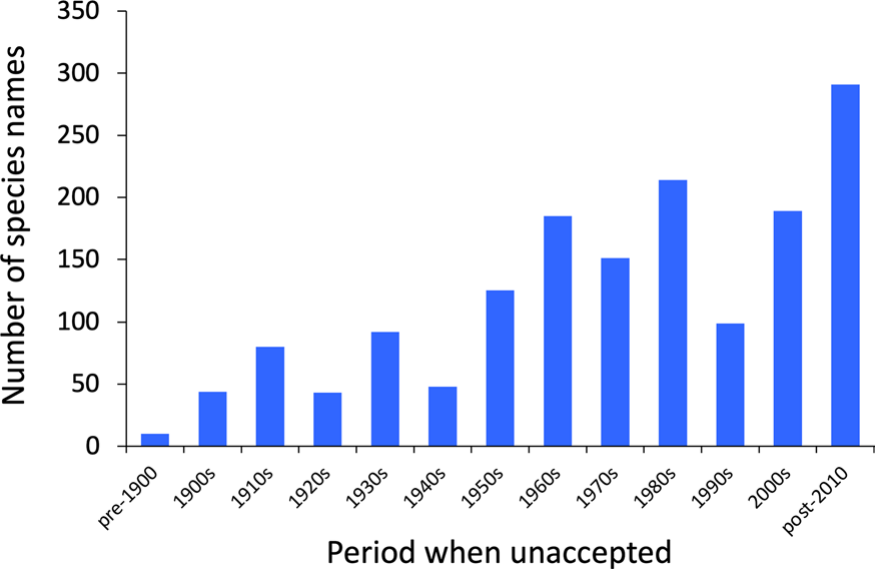
Number of helminth species names that became unaccepted per decade during the period covered by our dataset, for the 1571 species names (all four higher helminth taxa combined) for which data is available on the year when they were made unaccepted

Across all helminths combined, the last few decades have seen a greater number of species names being invalidated than the first several decades of the 20th century (Figure 3). There were fewer than 100 species binomial names being made unaccepted per decade from 1900 to 1950, while nearly 300 names have been invalidated since 2010.

**Fig. 4:**
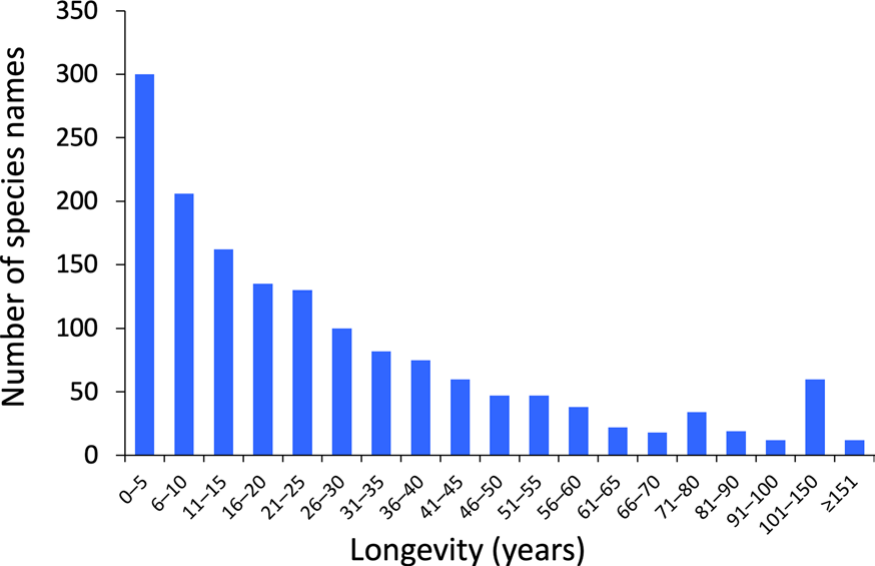
Frequency distribution of longevity (years) of Latin binomial names of helminth species, for the 1559 species names for which data is available on the year when they were made unaccepted (a further 12 species with negative longevity based on recorded data are excluded). The longevity of a species name corresponds to the number of years between the year when it was originally proposed and the year when it became unaccepted. Note the contraction of the scale towards the right on the x-axis

**Fig. 5:**
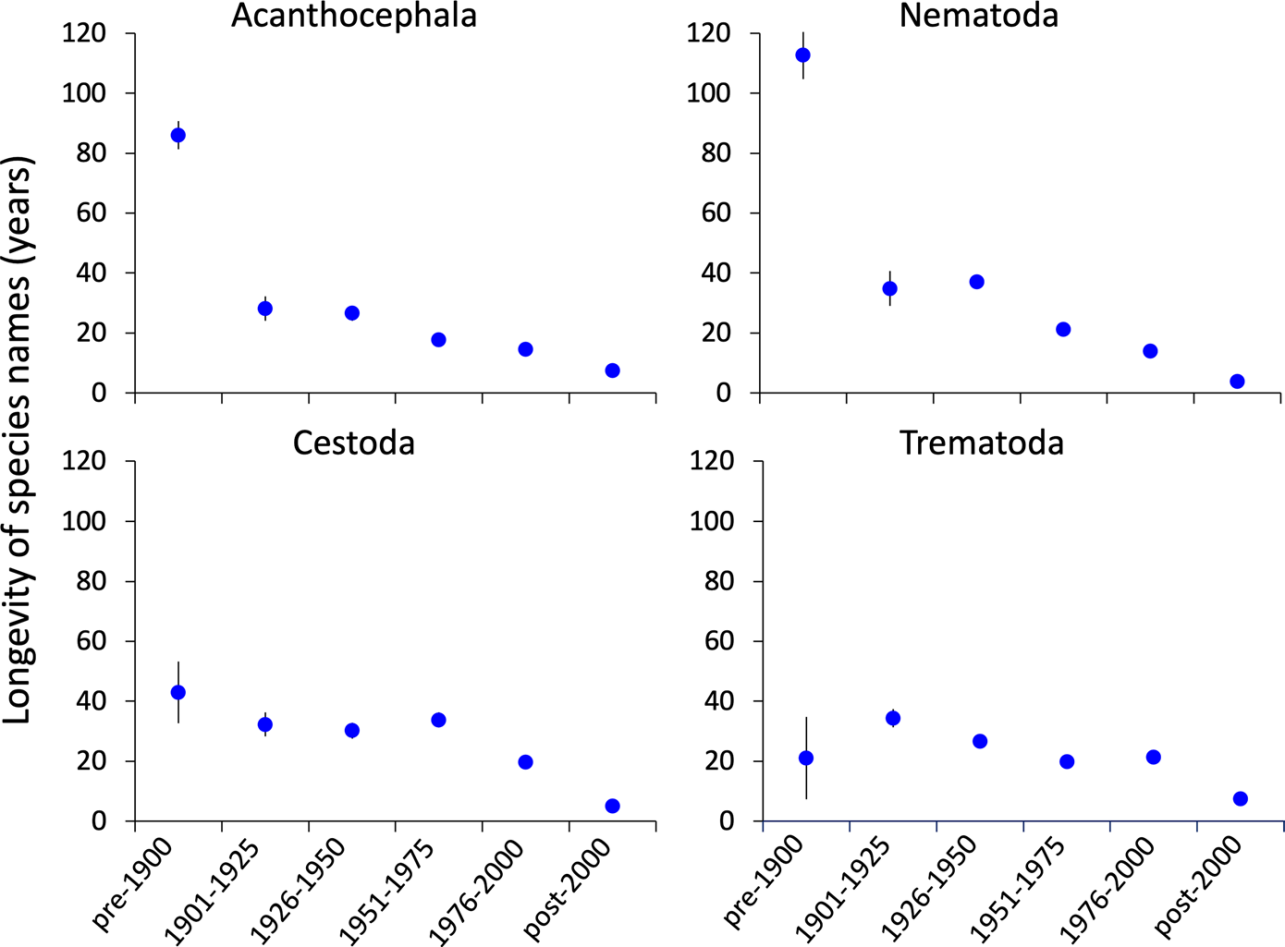
Mean (± standard error) longevity of Latin binomial names of species that have become unaccepted and are no longer valid (species with negative longevity based on recorded data are excluded). Data are shown separately for each of the four higher taxa of helminth parasites, and according to the time period in which they were first coined

Among all names that eventually became unaccepted, the one with the greatest longevity in the taxonomic record lasted 208 years before being invalidated, whereas several were unaccepted within one or two years after they were proposed, some even in the same year they were proposed. Overall, the average longevity of helminth species names that are currently unaccepted was 28.8 years. About 30% of names that are currently unaccepted were invalidated within 10 years of first being proposed, and about 50% within 20 years (Figure 4). On average, names of acanthocephalans and nematodes proposed in the 18^th^ and 19^th^ centuries that were eventually unaccepted remained in use for decades, often for over 100 years, before being invalidated, whereas those of cestodes and trematodes were unaccepted much faster (Figure 5). It must be pointed out that far fewer trematodes were described pre-1900 than for other helminth taxa, and therefore the estimates of the average longevity of unaccepted names for this group must be taken with caution. For all helminth taxa, species names proposed in the 20^th^ century that were eventually invalidated were generally unaccepted within 15-35 years (Figure 5). Those proposed after the year 2000 were the ones that lasted the fewest number of years before being unaccepted.

## Discussion

Earth’s natural ecosystems are facing a biodiversity crisis that some have labelled the sixth mass extinction (Ceballos et al. 2015; Cowie et al. 2022). However, even when species do not go extinct, sometimes their original Latin name does. Stable nomenclature not subject to frequent changes would provide biologists with standard and permanent species names that would facilitate communication. However, in spite of the adoption of codified sets of rules for naming living organisms overseen by international bodies (Winston 2018), newly coined species names are not forever. Regular taxonomic revisions combined with failures to recognise a previously known species cause many species names to eventually disappear from taxonomic inventories. Here, we provide a quantitative look at this phenomenon among helminth parasites. We must emphasise that our findings apply to the subset of species names included in our analysis, since our coverage extends to only the most speciose families of acanthocephalans, nematodes, cestodes and trematodes. However, we see no clear reason why these patterns should not apply across all helminth species.

Firstly, our results show that, overall, a substantial number of species names do not last forever. Indeed, about one-third of Latin binomial names proposed for helminth species in the past two-and-a-half centuries are no longer accepted. The nomenclature of helminth parasites is therefore far from stable over time. Not surprisingly, a higher proportion of names proposed a long time ago have since been unaccepted, compared to names proposed more recently, since there has been more time to re-evaluate the validity of older names. Out of all species names that are no longer accepted, about one third were unaccepted because they were found to be synonyms of previously described and named species. This means that of all helminth species described in the past two-and-a-half centuries, about one in nine (a third of a third) were not new species at all. Such a high level of synonymy complicates any attempt to estimate helminth biodiversity (Alroy 2002), an issue compounded by the often unrecognised cryptic diversity among helminth taxa (Poulin 2011; Pérez-Ponce de León and Poulin 2018). There have been some instances of mass synonymisation (“lumping”) of many species, such as multiple previously-named species in the cestode genus *Gangesia* being justifiably reduced to just a handful of valid species names (Ash et al. 2012). Avoiding synonyms would require that each new putative species be compared morphologically and genetically with all previously described species within the same genus or family, something that is practically impossible in most cases. However, recent descriptions of newly-discovered helminth species are more comprehensive and of greater quality than older ones. In particular, the number of previously named species to which newly described species are compared has risen significantly over the past decades (Poulin and Presswell 2016), suggesting that new species descriptions are generally much less likely to be later unaccepted as synonyms than species described many years ago.

Secondly, our data reveals that the number of species names that are being unaccepted annually has been increasing over time, peaking in the last decade. More species are being shifted to a different genus or synonymised with previously described ones than ever before. This indicates a recent rise in taxonomic activity aimed at revising the status of previously known species, a task greatly facilitated by the adoption of molecular tools in the past couple of decades. Over the same period, the number of new species descriptions of helminth parasites published annually has grown steadily, with the current annual output of new descriptions having doubled in the past 2-3 decades (Costello 2016; Poulin and Presswell 2016). In light of the demonstrated loss of taxonomic expertise currently threatening the field, as the number of active taxonomists appears to be dwindling (Poulin and Presswell 2022), the sustained high rates of species discovery and description, combined with high rates of taxonomic revision, are remarkable and a testimony to the work and tenacity of taxonomists.

Thirdly, most species names that were eventually unaccepted lasted only a few years after first being proposed, with their average longevity being about 29 years. In particular, names proposed since the year 2000 that were invalid for one reason or another were often unaccepted within 5 years. For example, multiple species in the trematode species *Opegaster* were transferred to the genus *Opecoelus* (Aken’Ova 2007), and then transferred back to *Opegaster* six years later (Bray and Justine 2013), quickly leading to many unaccepted binomial combinations. This illustrates the strong self-correcting nature of modern parasite taxonomy. However, several species names proposed in the 18th and 19th centuries persisted for well over 100 years before being unaccepted, in particular names of acanthocephalans and nematodes. There are undoubtedly many more invalid species names yet to be documented among the vast biodiversity of helminth parasites currently described and named.

Again, we must stress that these conclusions are based on the subset of species names included in our analysis, and not on all helminth species names ever published; yet, most likely these patterns apply broadly across all helminths. In addition, although we focused on binomial species names, higher taxonomic levels (genus, family, etc.) are also affected by changes of status. Taxonomic nomenclature is constantly evolving, and not only through the addition of new species to the known inventory of the planet’s biodiversity. Our analysis provides estimates of how many helminth species names proposed in the past two-and-a-half centuries turned out to be invalid, and how long on average it took for them to become unaccepted, based on when they were first proposed and the higher helminth taxon they belong to. These estimates may help to calibrate future attempts at predicting total parasite biodiversity, by allowing one to assign a probability of validity to each species name based on when it was proposed (Lessa et al. 2024). Our results also serve to illustrate in numbers the ability of taxonomic research to self-correct over time.

### Supplementary Information

Below is the link to the electronic supplementary material.

Supplementary file1 (XLS 1040 KB)

## Data Availability

The full dataset is available as Supplementary Material.
